# Ozagrel hydrochloride, a selective thromboxane A_2_ synthase inhibitor, alleviates liver injury induced by acetaminophen overdose in mice

**DOI:** 10.1186/1471-230X-13-21

**Published:** 2013-01-30

**Authors:** Yoshiro Tomishima, Yoichi Ishitsuka, Naoya Matsunaga, Minako Nagatome, Hirokazu Furusho, Mitsuru Irikura, Shigehiro Ohdo, Tetsumi Irie

**Affiliations:** 1 Department of Clinical Chemistry and Informatics, Graduate School of Pharmaceutical Sciences, Kumamoto University, 5-1 Oe-honmachi, Chuo-ku, Kumamoto, 862-0973, Japan; 2 Pharmaceutics Laboratory, Division of Clinical Pharmacy, Department of Medico-Pharmaceutical Sciences, Graduate School of Pharmaceutical Sciences, Kyushu University, 3-1-1, Maidashi, Higashi-ku, Fukuoka, 812-8582, Japan; 3 Center for Clinical Pharmaceutical Sciences, Faculty of Pharmaceutical Sciences, Kumamoto University, 5-1 Oe-honmachi, Chuo-ku, Kumamoto, 862-0973, Japan

## Abstract

**Background:**

Overdosed acetaminophen (paracetamol, N-acetyl*-p-*aminophenol; APAP) causes severe liver injury. We examined the effects of ozagrel, a selective thromboxane A_2_ (TXA_2_) synthase inhibitor, on liver injury induced by APAP overdose in mice.

**Methods:**

Hepatotoxicity was induced to ICR male mice by an intraperitoneal injection with APAP (330 mg/kg). The effects of ozagrel (200 mg/kg) treatment 30 min after the APAP injection were evaluated with mortality, serum alanine aminotransferase (ALT) levels and hepatic changes, including histopathology, DNA fragmentation, mRNA expression and total glutathione contents. The impact of ozagrel (0.001-1 mg/mL) on cytochrome P450 2E1 (CYP2E1) activity in mouse hepatic microsome was examined. RLC-16 cells, a rat hepatocytes cell line, were exposed to 0.25 mM N-acetyl*-p-*benzoquinone imine (NAPQI), a hepatotoxic metabolite of APAP. In this model, the cytoprotective effects of ozagrel (1–100 muM) were evaluated by the WST-1 cell viability assay.

**Results:**

Ozagel treatment significantly attenuated higher mortality, elevated serum alanine aminotransferase levels, excessive hepatic centrilobular necrosis, hemorrhaging and DNA fragmentation, as well as increase in plasma 2,3-dinor thromboxane B_2_ levels induced by APAP injection. Ozagrel also inhibited the hepatic expression of cell death-related mRNAs induced by APAP, such as jun oncogene, FBJ osteosarcoma oncogene (fos) and C/EBP homologous protein (chop), but did not suppress B-cell lymphoma 2-like protein11 (bim) expression and hepatic total glutathione depletion. These results show ozagrel can inhibit not all hepatic changes but can reduce the hepatic necrosis. Ozagrel had little impact on CYP2E1 activity involving the NAPQI production. In addition, ozagrel significantly attenuated cell injury induced by NAPQI in RLC-16.

**Conclusions:**

We demonstrate that the TXA_2_ synthase inhibitor, ozagrel, dramatically alleviates liver injury induced by APAP in mice, and suggest that it is a promising therapeutic candidate for the treatment of APAP-induced liver injury.

## Background

Acetaminophen (paracetamol, N-acetyl-*p*-aminophenol [APAP]) is a widely used analgesic/antipyretic drug with few side effects at therapeutic doses [1]. However, APAP overdose produces hepatic injury, and is the most frequent cause of acute liver failure in the United States [2], the United Kingdom [3] and other countries [4,5]. APAP preparations are often taken in excessive amounts for suicide, and the consumption of multiple-drug preparations containing APAP may also cause severe liver damage [6]. N-acetyl cysteine (NAC) is the only approved drug for treating APAP overdose. However, NAC has limited therapeutic efficacy against APAP hepatotoxicity. Consequently, a novel treatment approach is required.

Liver injury induced by APAP overdose manifests as extensive centrilobular necrosis, infiltration of inflammatory cells and bleeding. Massive production of cell death-related markers, such as cytokeratin 18 [7] and high mobility group box 1 protein [8], are also observed in serum of patients with APAP-induced liver injury. Hepatocellular necrosis is initiated by a reactive metabolite, N-acetyl-*p*-benzoquinone imine (NAPQI), mainly produced by cytochrome P450 (CYP) 2E1 [9-12]. NAPQI likely mediates injury via hepatic glutathione depletion, oxidative and nitrosative stress, and inflammation. Furthermore, the development and progression of liver injury induced by APAP appears to involve multiple mediators, including reactive oxygen species [13], peroxynitrite [14,15], cytokines [16-19] and eicosanoids [20,21].

Eicosanoids, such as prostaglandins (PGs) and thromboxanes, play an important role in the development of various diseases, as well as APAP hepatotoxicity [22-25]. North et al. [21] and Cavar et al. [26] demonstrated that PGE_2_ has a protective role in APAP-induced liver injury in zebrafish and mice, respectively. PGI_2_ (prostacyclin) also seems to act as a hepatoprotectant in APAP-induced liver injury [27]. In addition, Reilly et al. [28] suggested that cyclooxygenase (COX) 2-derived PGs, such as PGD_2_ and PGE_2_, have a hepatoprotective function in APAP-induced liver injury in mice. In contrast, thromboxane A_2_ (TXA_2_) seems to exacerbate APAP hepatotoxicity. Ketoconazole, OKY-1581 and benzyl imidazole, which inhibit TXA_2_ production, are able to prevent liver injury induced by APAP in rodents [20,29]. These observations suggest that inhibiting TXA_2_ production is a promising strategy for the treatment of liver injury due to APAP overdose. However, ketoconazole, originally an antifungal agent, has side effects caused by inhibition of CYPs [30]. In addition, while OKY-1581 and benzyl imidazole are frequently used in the laboratory, they are not approved for clinical use. Therefore, the prospect for the clinical use of TXA_2_ synthase inhibitors for the treatment of APAP-induced liver injury is unclear.

Ozagrel (OKY-046; (E)-3-[4-(imidazol-1-ylmethyl)phenyl]prop-2-enoic acid) was developed as a selective TXA_2_ synthase inhibitor and has been widely used for treating patients with bronchial asthma, and cerebral thrombosis and vasospasm in Japan [22,31,32]. Ozagrel alleviates the symptoms of various diseases as assessed by biochemical and clinical examination [22,23,31-33]. However, little has been reported on the effects of ozagrel on APAP hepatotoxicity.

In this study, we examined whether ozagrel could protect against APAP-induced liver injury in mice. We examined the effects of ozagrel on serum alanine aminotransferase (ALT) levels, mortality and histological changes induced by APAP treatment. In addition, we assessed hepatic glutathione content and the expression of cell death-related markers in the APAP treated mice, as well as CYP2E1 activity in mouse liver microsomes. Furthermore, we investigated the effects of ozagrel on NAPQI-induced hepatic injury *in vitro*.

## Methods

### Reagents

Ozagrel hydrochloride monohydrate was kindly donated by Ono Pharmaceutical CO., LTD. (Osaka, Japan) and Kissei CO., Ltd. (Nagano, Japan). APAP, NAPQI and NAC were purchased from Sigma-Aldrich (St. Louis, Missouri, USA). Metaphosphoric acid was purchased from Alfa Aesar (Ward Hill, Massachusetts, USA). Cell culture reagents were obtained from Gibco®-Life Technologies (Life Technologies Japan, Tokyo, Japan). HyClone™ fetal bovine serum (FBS) was purchased from Thermo Scientific (Logan, UT, USA). The cell counting kit and Cellstain® Double Staining Kit were obtained from Dojindo Laboratories (Kumamoto, Japan). All other reagents and solvents were of reagent grade. De-ionized and distilled bio-pure grade water was used throughout the study.

### Animal experiments

The APAP overdose-induced liver injury model was based on a model previously reported by our group [34]. Male 7–9-week-old ICR mice (Charles River Laboratories Japan INC., Yokohama, Japan) were used. Animals were housed in cages in a room under controlled conditions at 24°C with a 12-h light cycle, and given free access to food and water. Mice were fasted overnight, but given access to water, prior to experiments. All experimental procedures conformed to the animal use guidelines of the committee for Ethics on Animal Experiments of Kumamoto University (approval numbers C23-269 and C22-172).

Mice were divided into the following groups: (1) vehicle group, phosphate buffered saline (PBS) + saline treatment; (2) APAP group, APAP (330 mg/kg) + saline treatment; (3) APAP + ozagrel group, APAP (330 mg/kg) + ozagrel (100 or 200 mg/kg) treatment; (4) APAP + NAC group, APAP (330 mg/kg) + NAC (600 mg/kg). APAP was dissolved in warmed PBS (55°C). Ozagrel and NAC were dissolved in saline and administered intraperitoneally 30 min after the APAP injection. Mice were euthanized 4 h after APAP injection, and blood and liver samples were collected. In the survival study, mice were monitored for 48 h after the APAP injection.

### Serum alanine transaminase level

Blood samples were collected from the inferior vena cava and immediately centrifuged at 4000 × g at 4°C for 10 min, and sera were collected. Sera were stored at −30°C until assay. Serum ALT levels were determined using commercial assay kits (Wako Pure Chemical Industries, Ltd., Osaka, Japan).

### Hepatic histopathology

Liver tissue samples were fixed in 10% neutral buffered formalin and then embedded in paraffin. Microtome sections, 3-μm-thick, were prepared and stained with hematoxylin-eosin (H&E). Histological scoring was performed using a modification of a previously reported method [35,36]. Ten random fields for each section were analyzed by light microscopy. Liver injury was scored as follows: 0, no damage; 1, only a few fields affected; 2, zonal necrosis in most fields; 3, at least 3 foci of zonal necrosis in all fields; 4, centrilobular necrosis in most lobes in all fields; 5, panlobular confluent necrosis and hemorrhaging in all fields. In addition, to evaluate hepatic cell death, terminal deoxynucleotidyl transferase dUTP nick end labeling (TUNEL) staining was performed using the ApopTag® Peroxidase In Situ Apoptosis Detection Kit (Merck Millipore, Billerica, MA, USA) as described in the manufacturer’s instructions.

### Plasma 2,3-dinor thromboxane B_2_ level

Plasma samples were collected in ethylenediaminetetraacetic acid containing blood collection tubes (Becton Dickinson and Company Japan Inc., Tokyo, Japan) to which indomethacin (10 μM) (Sigma) was added, and centrifuged (3000 × g at 4°C for 10 min). Plasma level of 2, 3-dinor thromboxane B_2_ (2,3-dinor TXB_2_), a stable metabolite of TXA_2_, was determined using enzyme immunoassay kit (Cayman, Michigan, USA) as described in the manufacturer’s instructions.

### Hepatic RNA isolation and quantitative real-time RT-PCR analysis

Liver tissue samples were weighed and cooled, and stored in liquid nitrogen until assay. To obtain total RNA, liver samples were homogenized in TRIzol® reagent (Invitrogen™-Life Technologies Japan, Tokyo, Japan) according to the manufacturer’s instructions. Quantitative real-time RT-PCR for mouse *Jun* oncogene (*Jun*; NM_010591), FBJ osteosarcoma oncogene (*Fos*; NM_010234), C/EBP homologous protein (*Chop*; NM_007837), B-cell lymphoma 2-like protein11 (*Bim*; NM_207680) and beta actin (NM_007393) was carried out using the following primers: *Jun* sense, ATCCACGGCCAACATGCTC and antisense, ACGTTTGCAACTGCTGCGTTAG; *Fos* sense, TTACGCCAGAGCGGGAATG and antisense, GTTCCCTTCGGATTCTCCGTTT; *CHOP* sense, AGCTGGAAGCCTGGTATGAGGA and antisense, AGCTAGGGACGCAGGGTCAA; *Bim* sense, CCGGAGATACGGATTGCACAG and antisense, CAGCCTCGCGGTAATCATTTG. Synthesis of cDNA from hepatic total RNA was performed using the High Capacity cDNA Reverse Transcription Kit (Applied Biosystems-Life Technologies Japan, Tokyo, Japan). Real-time PCR analysis was performed on diluted cDNA samples with Fast SYBR® Green master mix (Applied biosystems-Life Technologies Japan) using StepOnePlus™ Real-time PCR system (Applied biosystems-Life Technologies Japan). Melting curve analysis was performed for validation of specific amplification. The relative quantity of target gene mRNA was normalized against the beta actin level (internal control) and expressed as fold induction.

### Hepatic total glutathione content

The liver tissue samples were weighed and stored at −80°C until assay. Tissue homogenates were prepared in 5% metaphosphoric acid solution at a 1:5 (w/v) ratio and centrifuged for 10 min at 1000 × g at 4°C. The supernatant was collected and total GSH concentration was measured using a BIOXYTECH GSH/GSSG-412 (OXIS Health Products, Inc, Portland, OR) according to the manufacturer’s protocol. GSH content was expressed as nmol/mg tissue.

### CYP2E1 activity

CYP2E1 activity in mouse liver was examined using a method previously reported by our laboratory with minor modification [34]. In brief, a microsomal fraction from the liver of the ICR mouse was prepared according to a previously reported method [37]. CYP2E1 activity was evaluated by conversion by the enzyme of the Vivid® Blue Substrate (Invitrogen™-Life Technologies) into a fluorescent metabolite. Microsomes from mouse liver, NADPH-cytochrome P450 reductase, and cytochrome b5 and NADPH regeneration system were mixed and incubated with or without varying concentrations of ozagrel (0.001–1.0 mg/mL). The fluorescence intensity of the probe at excitation and emission wavelengths of 409 and 460 nm, respectively, were measured using a fluorescence microplate reader. CYP2E1 activity was then expressed as the fluorescence intensity/mg protein.

### Cell culture and measurements of cell viability

RLC-16 cells, a rat hepatocyte cell line, were purchased from RIKEN BioResource Center (Ibaraki, Japan). Cells were maintained under 5% CO_2_ and 95% air at 37°C in MEM with 10% FBS, 100 IU/ml penicillin and 100 μg/mL streptomycin. The cell injury induced by NAPQI was evaluated in accordance with methods described previously [38,39]. In brief, RLC cells were seeded 1 × 10^4^ cells/well into a 96 well plate. After 24 h to allow cells to adhere, the medium was replaced with fresh medium containing 250 μM NAPQI, with or without ozagrel (1–100 μM). Cell viability was estimated 24 h after NAPQI addition by measuring mitochondrial dehydrogenase activity with a modified MTT assay, namely, the water-soluble tetrazolium salt (WST-1) assay, using a kit (Dojindo Laboratories, Kumamoto, Japan).

### Statistical analysis

Results are expressed as mean ± S.E.M. Statistical analysis was performed using GraphPad Prism ver. 5.01 (GraphPad Software, San Diego, CA). Multiple comparisons were made to examine the statistical significance of the results. When uniform variance of the result was identified by Bartlett’s analysis (*p* < 0.05), one-way analysis of variance was used to test for statistical differences. When significant differences (*p* < 0.05) were identified, the results were further analyzed by Dunnett’s or Tukey’s multiple range test for significant differences among the values. If uniform variance of the result was not identified, non-parametric multiple comparisons were made. After confirming significant differences (*p* < 0.05) using Kruskal-Wallis analysis, the differences were then examined by applying Dunnett’s test. Analysis of histological score was also performed using these non-parametric multiple comparison tests. As for comparisons of two unpaired values, unpaired Student’s *t*-test was performed. Survival data were analyzed using the Kaplan-Meier method, and the log-rank test was used to compare statistical significances.

## Results

### Effects on histological changes, elevation of serum ALT level and survival rate induced by APAP injection in mice

**Figure 1:**
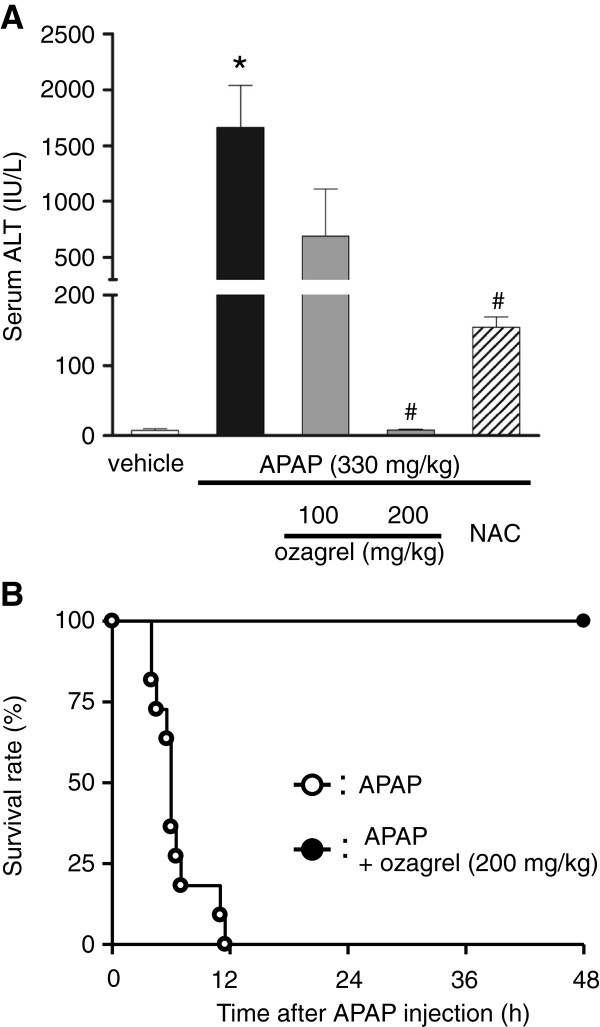
**Effects of ozagrel on serum ALT level and survival rate in APAP-induced liver injury. **(**A**) Serum ALT levels 4 h after the APAP injection. Mice were treated with ozagrel (100 or 200 mg/kg), NAC (600 mg/kg) or saline 30 min after the APAP (330 mg/kg) injection. There was a significant increase in the APAP group (black bar) compared with the vehicle group (white bar). The increase in serum was significantly reduced by ozagrel (APAP + ozagrel group; gray bar) and NAC (APAP + NAC group; hatched bar). Each bar represents the mean ± S.E.M. (n = 5–7). *p < 0.01 compared with the vehicle group, ^#^*p* < 0.01 compared with the APAP group. (**B**) Survival rate over a 48-h period in mice after APAP injection. Mice were treated with ozagrel (200 mg/kg, n=5) or saline (n=11) 30 min after the APAP (330 mg/kg, n=16) injection. A significant difference (*p* = 0.004) was observed between the APAP group and the APAP + ozagrel group.

We examined changes in ALT level in serum 4 h after treatment with APAP (330 mg/kg), as a measure of hepatic damage. APAP caused a significant increase in serum ALT level compared with the vehicle group (Figure 1A). In the APAP + ozagrel (100 or 200 mg/kg) group, ALT level was significantly reduced compared with the APAP group and the APAP + NAC (600 mg/kg) group. ALT level (7.8 ± 1.4 IU/L) in the APAP + ozagrel (200 mg/kg) group was similar to that (7.4 ± 2.4 IU/L) in the vehicle group. As shown in Figure 1B, all mice died within approximately 12 h after APAP injection. In contrast, all of the mice in the APAP + ozagrel (200 mg/kg) group survived.

**Figure 2:**
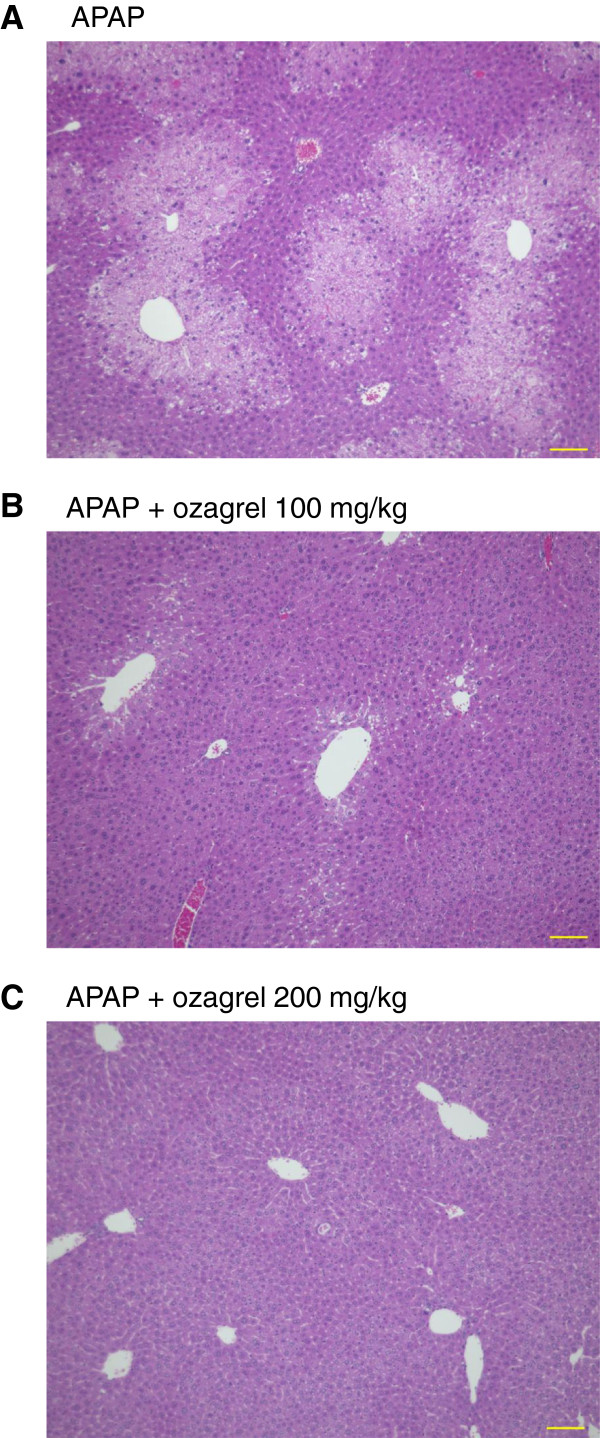
**Effect of ozagrel on hepatic histopathological changes induced by APAP injection.** Representative hepatic sections stained with H&E are shown. Mice were treated with ozagrel (100 or 200 mg/kg) or saline 30 min after the APAP (330 mg/kg) injection. (**A**) APAP group; (**B**) APAP + ozagrel (100 mg/kg) group; and (**C**) APAP + ozagrel (200 mg/kg) group. Scale bar: 100 μm.

Representative H&E staining of histological section is shown in Figure 2. Severe centrilobular necrosis, hemorrhaging and hepatocyte degeneration were observed in the APAP group. These pathological changes induced by APAP were strongly suppressed in livers of mice in the APAP + ozagrel (200 mg/kg) group. Minor hepatic pathological changes were observed in the APAP + ozagrel (100 mg/kg) group, though severe centrilobular necrosis was observed in a single mouse. The histopathological scores in the APAP + ozagrel (200 mg/kg) group were significantly lower (p < 0.01) than in the APAP group (Table 1).

**Table 1:** Histopathological scores in mice treated with ozagrel 30 min after APAP (330 mg/kg) injection

**Treatment**	**Histological scores (4 h after the APAP injection)**					
	**0**	**1+**	**2+**	**3+**	**4+**	**5+**
APAP + saline	0	0	0	2	1	2
APAP + ozagrel 100 mg/kg	2	0	2	0	0	1
APAP + ozagrel 200 mg/kg	4	1	0	0	0	0

Mice were treated with ozagrel (100 or 200 mg/kg) or saline 30 min after the APAP (330 mg/kg) injection. Hepatic sections from mice 4 h after APAP injection were scored according to severity of injury: 0, no damage; 1, only a few fields affected; 2, zonal necrosis in most fields; 3, at least 3 foci of zonal necrosis in all fields; 4, centrilobular necrosis in most lobes in all fields; 5, panlobular confluent necrosis and hemorrhaging in all fields. Values represent the total number of mice given each score (n = 5).

### Changes in plasma 2, 3-dinor TXB_2_

**Figure 3:**
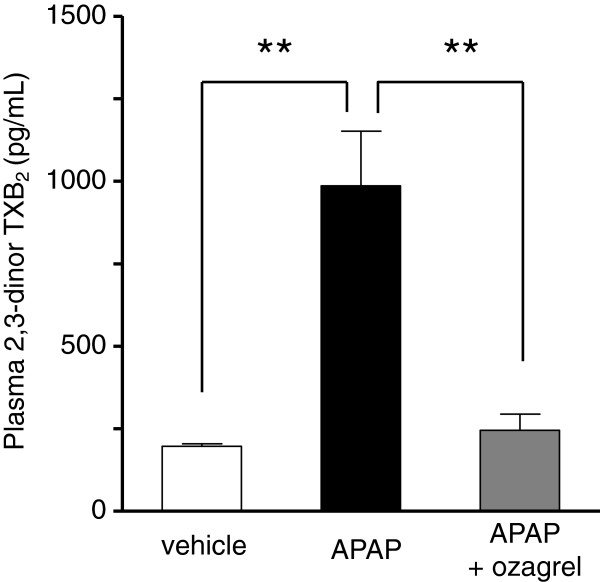
**Effect of ozagrel on plasma 2,3-dinor TXB_2_ levels in APAP-induced liver injury.** Mice were treated with ozagrel (200 mg/kg) or saline 30 min after the APAP (330 mg/kg) injection. Plasma 2,3-dinor TXB_2_ levels were measured by enzyme immunoassay 4 h after the APAP injection. There was a significant increase in plasma 2,3-dinor TXB_2_ in the APAP group (black bar) compared with the vehicle group (white bar). The increase was significantly suppressed by ozagrel (the APAP + ozagrel group; gray bar). Each value represents the mean ± S.E.M. (n = 6). **p < 0.01.

As shown in Figure 3, plasma 2, 3-dinor TXB_2_ level was significantly increased by injection of APAP, and the level in the APAP group was approximately 5-fold greater than in the vehicle group. An increase in 2, 3-dinor TXB_2_ level was not observed in the APAP + ozagrel group.

### Effects on DNA fragmentation and hepatic mRNA expression induced by APAP

**Figure 4:**
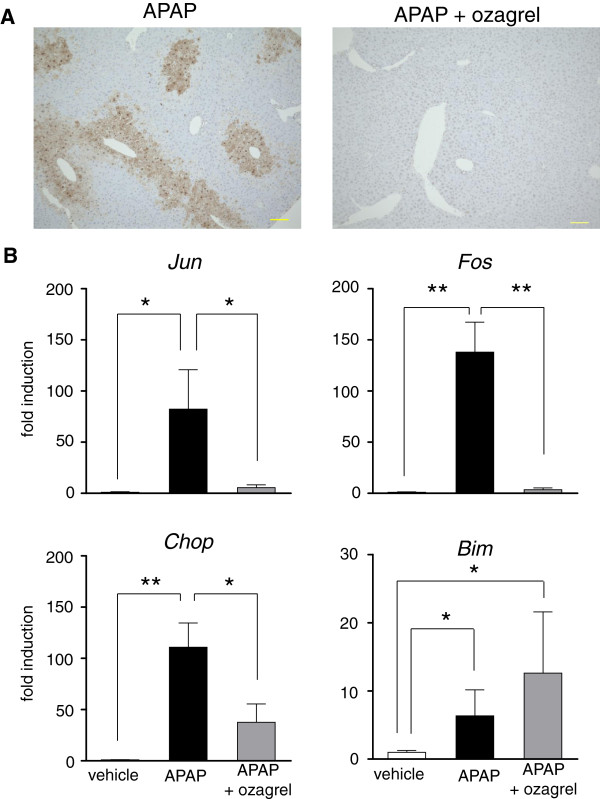
**Effects of ozagrel on hepatic cell death-related markers in liver induced by APAP injection.** The effects of ozagrel on DNA fragmentation (**A**) and cell death-related gene expression (**B**) in liver induced by APAP injection. Mice were treated with ozagrel (200 mg/kg) or saline 30 min after the APAP (330 mg/kg) injection. The liver tissue samples were collected 4 h after the APAP injection. (**A**) Representative TUNEL staining. A significant number of brown TUNEL-positive cells were observed in the APAP group, while only a few brown cells were observed in the APAP + ozagrel group. (**B**) Changes in mRNA levels of *Jun*, *Fos*, *chop* and *Bim* in liver were analyzed by quantitative real-time RT-PCR. There were significant increases in the relative expression levels of all of these genes in the APAP group (black bar) compared with the vehicle group (white bar). The increases in *Jun*, *Fos* and *chop* mRNA levels, but not that of *Bim* mRNA, were significantly reduced by ozagrel (APAP + ozagrel group; gray bar). Each value represents the mean ± S.E.M. (n = 5–6). *p < 0.05, **p < 0.01.

To investigate the effects of ozagrel on nuclear DNA fragmentation, the TUNEL assay was performed, and to examine the expression of cell death-related genes, quantitative real-time RT-PCR was performed for mouse liver *Jun*, *Fos*, *Chop* and *Bim* transcripts. Representative TUNEL staining is shown in Figure 4A. Numerous TUNEL-positive cells were observed in APAP treated mice. In contrast, only a few positive cells were visible in the APAP + ozagrel group.

As shown in Figure 4B, APAP caused significant increases in *Jun*, *Fos*, *Chop* and *Bim* mRNA expression compared with vehicle treatment. The increases in *Jun*, *Fos* and *Chop* expression induced by APAP were inhibited by ozagrel (200 mg/kg), while *Bim* expression was not attenuated.

### Changes in liver glutathione content and CYP 2E1 activity in hepatic microsomes

**Figure 5:**
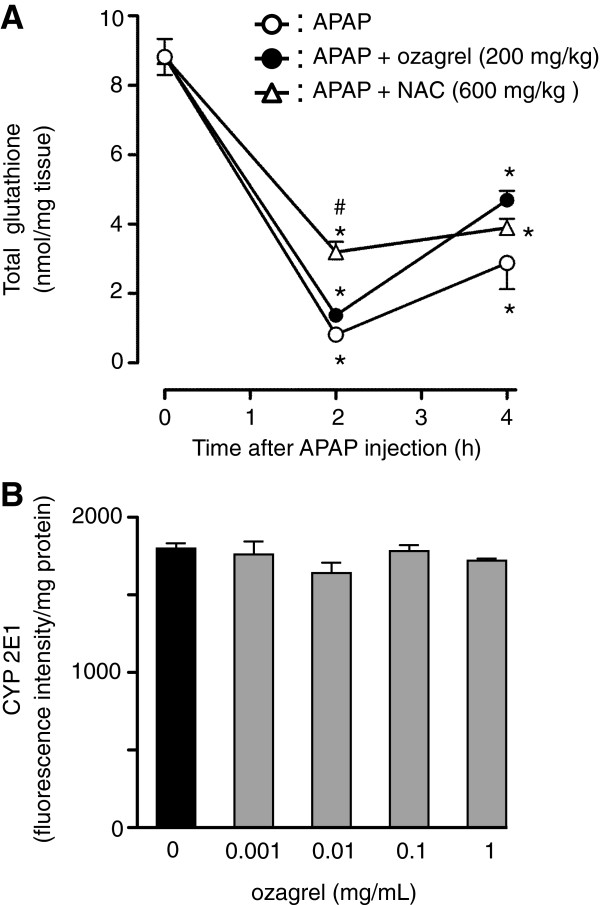
**Effects of ozagrel on hepatic GSH depletion induced by APAP and CYP 2E1 level. **(**A**) Mice were treated with ozagrel (200 mg/kg), NAC (600 mg/kg) or saline 30 min after the APAP (330 mg/kg) injection. The liver tissue samples were collected 0, 2 and 4 h after the APAP injection. Significant decreases in hepatic total GSH level were observed in all groups. The GSH level of APAP + NAC group was significantly higher than that of APAP group and APAP + ozagrel group at 2 h. Each value represents the mean ± S.E.M. (n = 4–5). *p < 0.001 compared with the 0 h group. ^#^*p* < 0.001 compared with the APAP group and APAP + ozagrel group. (**B**) CYP 2E1 level in mouse hepatic microsomes was evaluated using a fluorogenic probe, the Vivid® CYP2E1 Blue Substrate. Ozagrel at various concentrations (0.001–1.0 mg/mL) was incubated with the reaction mixture and the fluorescence intensity of the fluorescent metabolite was measured. Each value represents the mean ± S.E.M. (n=3). There were no significant differences between the groups.

To examine the mechanisms underlying the protective effect of ozagrel against APAP-induced liver injury, hepatic GSH consumption and mouse CYP 2E1 activity were assessed. As shown in Figure 5A, hepatic total GSH level 2 and 4 h after APAP injection were significantly decreased in all groups compared with the value at 0 h. No significant difference was observed between these values in the APAP group and the APAP + ozagrel group. In contrast, the APAP + NAC group had significantly higher GSH levels compared with the APAP and APAP + ozagrel groups at 2 h.

The results of the *in vitro* CYP2E1 activity assay are shown in Figure 5B. Ozagrel, at any dose (0.001–1.0 mg/mL), did not inhibit CYP2E1 activity.

### Protective effect against NAPQI-induced cell death

**Figure 6:**
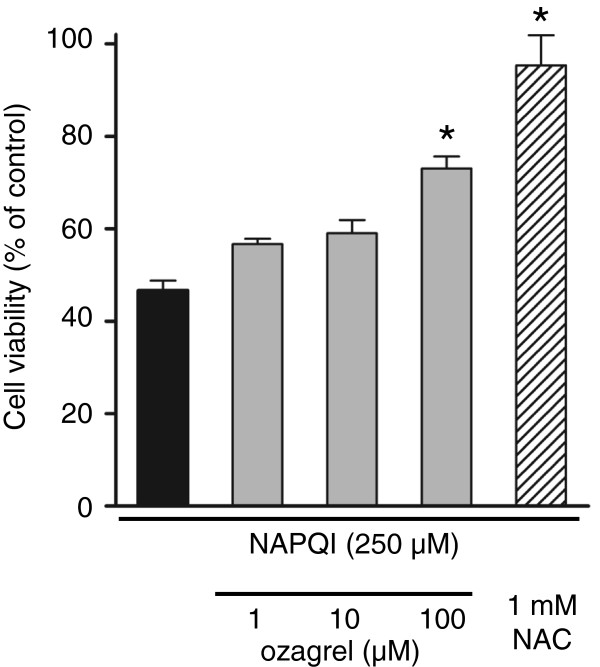
**The effect of ozagrel on cell viability reduced by NAPQI in RLC-16 cells.** Cells were exposed to 250 μM NAPQI in either the presence or absence of ozagrel (1–100 μM) or NAC (1 mM). Cell viability was measured 24 h after the NAPQI exposure using the WST-1 assay. NAPQI (black bar) induced a decrease in cell viability. This reduction was significantly inhibited by ozagrel (gray bar) and NAC (hatched bar). Each bar represents the mean ± S.E.M. (n = 4). *p < 0.01 compared with the APAP group.

As shown in Figure 6, RLC-16 cells treated with 250 μM NAPQI for 24 h showed an approximately 50% decrease in cell viability. When 1–100 μM ozagrel was added to the culture medium immediately after NAPQI exposure, the decrease in cell viability was significantly inhibited in a dose-dependent manner. Treatment with NAC (1 mM) also significantly attenuated the reduction in cell viability (Figure 6).

## Discussion

We demonstrated that the APAP-induced increases in serum ALT and plasma 2,3-dinor TXB_2_ levels, as well as the rise in mortality rate, were drastically attenuated by ozagrel, a selective TXA_2_ synthase inhibitor, administered 30 min after the APAP injection. In addition, the histopathological changes produced by APAP were also suppressed by ozagrel. These results indicate that ozagrel protects against hepatotoxicity induced by APAP. The protective effect of ozagrel was comparable to that of NAC, the sole antidote for APAP hepatotoxicity.

A number of animal studies have demonstrated that ozagrel is protective against various forms of trauma and disease, including lung injury [23,33], bronchial asthma [22,40] and ischemia/reperfusion-induced organ injury [41,42]. Ozagrel promptly inhibits TXA_2_ synthase *in vitro* and *in vivo*[31]. In our previous study, we demonstrated that ozagrel protects against acute lung injury induced by fat embolism in guinea pigs [33]. It is notable that ozagrel was protective despite being administered 60 min after APAP injection (Additional file 1: Figure 1S). From a clinical perspective, a drug that has efficacy when administered *after* the initiating insult has tremendous therapeutic potential.

Various inflammatory mediators are thought to be involved in the development of liver injury induced by APAP [19], and TXA_2_ appears to be one of these [20,29]. In this study, we observed a significant increase in plasma 2,3-dinor TXB_2_ levels following APAP injection, which is in agreement with previous reports. Reilly et al. (2001) [28] observed significantly elevated APAP-induced hepatotoxicity in COX-2 deficient mice and in mice treated with a COX-2 inhibitor. These authors suggested that eicosanoids, such as PGE and PGI_2_, have an important hepatoprotective function, and that COX inhibition may exacerbate APAP-induced liver injury. However, the excessive production of 2,3-dinor TXB_2_ induced by APAP and the protective effects of ozagrel observed in this study suggest that TXA_2_ is an aggravating factor in APAP-mediated hepatotoxicity.

*Jun* and *Fos* have been reported to be associated with the degree of APAP-induced liver injury [43,44]. In this study, ozagrel significantly suppressed the APAP-induced elevation in hepatic *Jun* and *Fos* mRNA expression. This result provides further support for a hepatoprotective function for ozagrel. In addition, APAP significantly induced expression of *Chop* and *Bim* mRNA, both of which play important roles in cell death during endoplasmic reticulum stress in various diseases. Nagy et al. (2007) [45] observed DNA fragmentation and CHOP induction in the livers of APAP-treated mice. We demonstrated that ozagrel attenuates the increase in the number of TUNEL-positive cells and suppresses the elevation in *Chop* mRNA expression induced by APAP in the liver. However, ozagrel did not repress the APAP-mediated increase in *Bim* mRNA expression. Badmann et al. (2011) [46] reported that *Bim*-deficient mice were substantially protected from APAP-induced liver damage, and suggested that *Bim* plays an important role in the development of liver injury induced by APAP. In cell death-related processes, the *Bim* pathway seems to be regulated not only by the transcriptional activation of *Bim*[46] but also by other mechanisms, such as phosphorylation or proteasomal degradation of *Bim* protein [47] and binding to anti-apoptotic molecules, including Bcl-2 and Bcl-XL [48]. Therefore, the effects of ozagrel on the *Bim* pathway remain unclear, and further study is needed to fully elucidate the effects of the drug. In this context, our finding that ozagrel does not affect APAP-induced *Bim* mRNA expression (in contrast to *Chop*, *Jun* and *Fos* mRNAs) is interesting and provides insight into the mechanisms underlying the protective effect of ozagrel against APAP hepatotoxicity.

The hepatotoxicity of APAP is triggered by a reactive metabolite, NAPQI, which is generated mainly by CYP2E1 [9-12]. Jaeschke et al. (2012) [19] and Bantel and Schulze-Osthoff (2012) [49] found that excessive NAPQI production depletes the hepatic GSH, and that this process is critical for the initiation of APAP hepatoxicity. In this study, ozagrel did not significantly attenuate the reduction in hepatic GSH content induced by APAP. In addition, ozagrel did not inhibit CYP2E1 activity in liver microsomes. These results suggest that the protective effect of ozagrel against APAP-induced hepatic injury is not due to inhibition of NAPQI production. This notion is supported by the *in vitro* results showing that ozagrel attenuates cellular injury induced by NAPQI in the RLC-16 hepatocyte cell line. These results suggest that the target of ozagrel, TXA_2_ synthase, may be situated downstream of NAPQI production and may play important roles in the development of APAP-induced liver injury. However, further detailed study (e.g., using TXA_2_ receptor knockout mice and TXA_2_ synthase knockdown) is required to fully uncover the roles of TXA_2_ in APAP hepatotoxicity.

OKY-1581 ((E)-2-methyl-3-[4-(pyridin-3-ylmethyl)phenyl]prop-2-enoic acid) was discovered as a selective inhibitor of TXA_2_ synthase, along with ozagrel, and shows protective effects against APAP hepatotoxicity in mice [29]. However, the development of clinical OKY-1581 formulations has been abandoned because of adverse reactions observed in clinical trials. In comparison, ozagrel was found to be an ideal compound for use as a highly selective TXA_2_ synthase inhibitor [31], and it is in clinical usein Japan. Although further studies to evaluate the usefulness and safety of ozagrel in patients with APAP hepatotoxicity are needed, the results of this study suggest that the inhibition of TXA_2_ synthase by the drug is effective for the treatment of APAP-induced liver injury.

NAC is clinically used as an antidote for APAP intoxication, and it is thought that NAC provides cysteine, which is a precursor of GSH (which traps NAPQI), leading to a decrease in toxicity [16,50]. In this study, NAC increased hepatic GSH content 2 h after the APAP injection and significantly prevented cell injury induced by NAPQI in RLC-16 cells. These results indicate that NAC provides GSH and detoxifies NAPQI. In comparison, although ozagrel exerted a remarkable hepatoprotective action against APAP-induced liver injury in mice, GSH content 2 h after APAP injection was not increased by the drug. These results indicate that ozagrel has a mode of action different from that of NAC in protection against APAP hepatotoxicity. For the development of new therapeutic strategies, it is interesting that ozagrel has a mechanism of action distinct from that of an existing agent, NAC.

Although the protective effect of ozagrel against cellular injury induced by NAPQI in RLC-16 cells was less robust than that of NAC, it may not indicate the inferiority of ozagrel as a therapeutic agent for APAP hepatotoxicity. The *in vitro* model of APAP hepatotoxicity using the cell culture system does not seem to fully agree with the *in vivo* model. For example, although the c-Jun N-terminal kinase inhibitor SP600125 drastically attenuates APAP-induced liver injury in the *in vivo* model, it has little effect in an *in vitro* model [44,51]. Therefore, the *in vitro* model of APAP hepatotoxicity may not be adequate for comparison of the efficacy of drugs. Nonetheless, it may provide insight into the mechanisms underlying the protective effect of the agents against APAP liver injury. If ozagrel protects against APAP-induced liver injury only through the modulation of inflammatory cell activity, such as inhibition of neutrophils or Kupffer cells, it would not be able to exert a protective action in an *in vitro* model. Therefore, the results of the *in vitro* model demonstrate that ozagrel, at least in part, protects against APAP hepatotoxicity by inhibiting the oncotic necrosis of hepatocytes.

## Conclusion

In summary, we demonstrate that the TXA_2_ synthase inhibitor ozagrel strikingly ameliorates liver injury induced by APAP in mice. We suggest that ozagrel is a promising candidate for the treatment of hepatotoxicity due to accidental or intentional APAP overdose.

## Competing interests

The authors declare that they have no competing interests. This work was supported by a Grant-in-Aid for Scientific Research for Young Scientists (B), No. 21790524 and 23790603 to Ishitsuka Y.

## Authors’ contributions

YT & YI designed research. YT, YI, NM, MN & HF performed research. YT, YI, MI & TI analyzed the data. YT, YI, MI, SO and TI drafted the manuscript. All authors read and approved the final manuscript.

## Pre-publication history

The pre-publication history for this paper can be accessed here:

http://www.biomedcentral.com/1471-230X/13/21/prepub

## Supplementary Material

Additional file 1 **Figure 1S. **Survival rate over a 48-h period in mice after APAP injection. Mice were treated with ozagrel (200 mg/kg), NAC (600 mg/kg) or saline 60 min after the APAP (330 mg/kg) injection. A significant difference (*p* = 0.001) was observed between the APAP group and the APAP + ozagrel group (n = 12–17). Click here for file
