# Supplementary material for: Ozagrel hydrochloride, a selective thromboxane A2 synthase inhibitor, alleviates liver injury induced by acetaminophen overdose in mice
Source: BMC Gastroenterol. 2013 Jan 30;13:21. doi: 10.1186/1471-230X-13-21 (PMC3568068; doi:10.1186/1471-230X-13-21)
Supplement: Additional file 1 — Figure 1S. Survival rate over a 48-h period in mice after APAP injection. Mice were treated with ozagrel (200 mg/kg), NAC (600 mg/kg) or saline 60 min after the APAP (330 mg/kg) injection. A significant difference (p = 0.001) was observed between the APAP group and the APAP + ozagrel group (n = 12–17). [file 1471-230X-13-21-S1.ppt]

## Slide 1
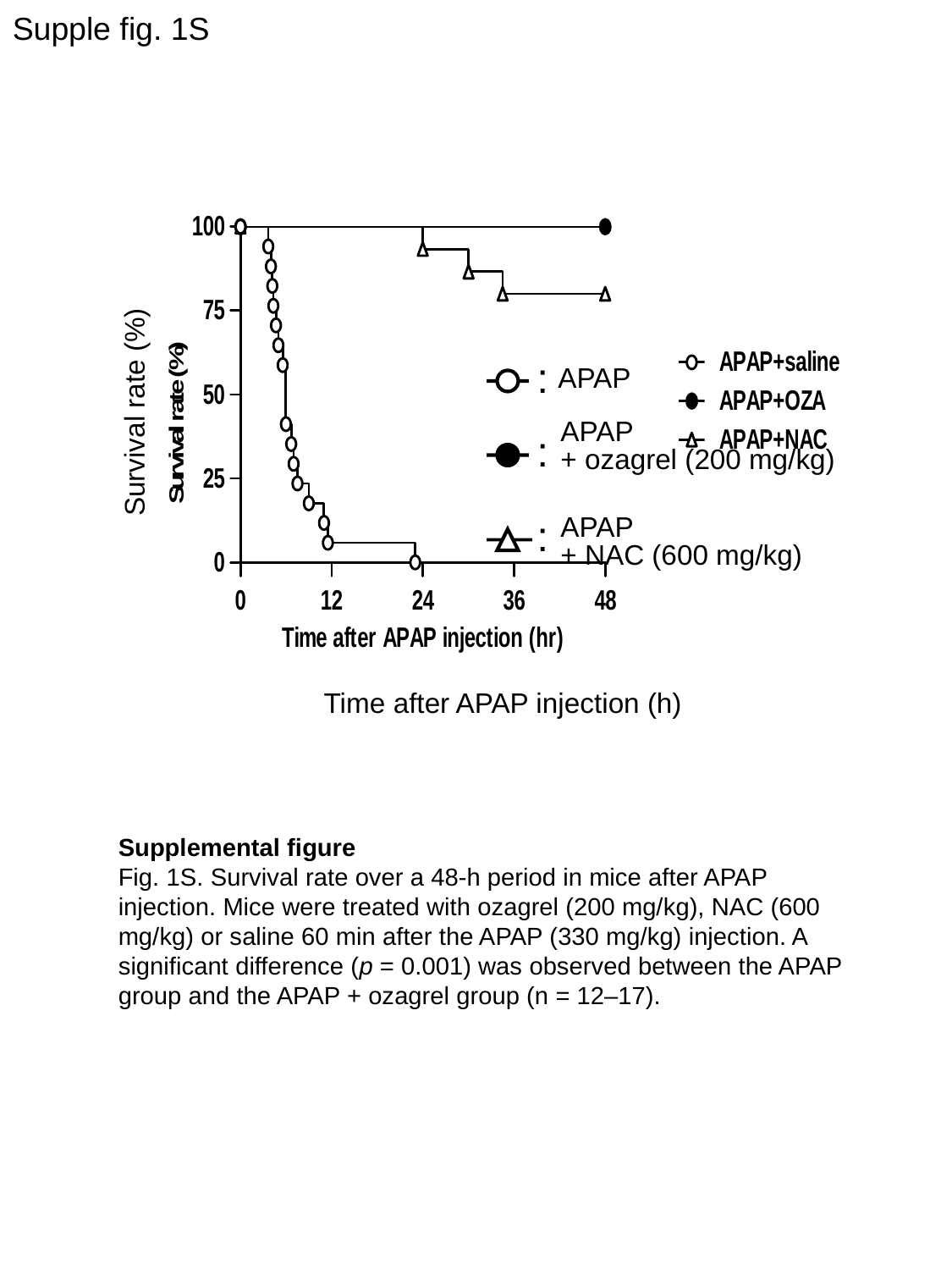

Supple fig. 1S
:
APAP
Survival rate (%)
APAP
+ ozagrel (200 mg/kg)
:
:
APAP
+ NAC (600 mg/kg)
Time after APAP injection (h)
Supplemental figure
Fig. 1S. Survival rate over a 48-h period in mice after APAP injection. Mice were treated with ozagrel (200 mg/kg), NAC (600 mg/kg) or saline 60 min after the APAP (330 mg/kg) injection. A significant difference (p = 0.001) was observed between the APAP group and the APAP + ozagrel group (n = 12–17).
